# Prevalence of Strongyloidiasis in a Cohort of Migrants in Italy and Accuracy of a Novel ELISA Assay for *S. stercoralis* Infection, a Cross-Sectional Study

**DOI:** 10.3390/microorganisms9020401

**Published:** 2021-02-15

**Authors:** Dora Buonfrate, Rosalia Marrone, Ronaldo Silva, Concetta Mirisola, Andrea Ragusa, Manuela Mistretta, Francesca Perandin, Zeno Bisoffi

**Affiliations:** 1 Department of Infectious Tropical Diseases and Microbiology (DITM), IRCCS Sacro Cuore Don Calabria Hospital, 37024 Negrar, Italy; ronaldo.silva@sacrocuore.it (R.S.); andrea.ragusa@sacrocuore.it (A.R.); manuela.mistretta@sacrocuore.it (M.M.); francesca.perandin@sacrocuore.it (F.P.); zeno.bisoffi@sacrocuore.it (Z.B.); 2 National Institute for Health Migration and Poverty (INMP), 00153 Rome, Italy; rosalia.marrone@inmp.it (R.M.); concetta.mirisola@inmp.it (C.M.); 3 Department of Diagnostics and Public Health, University of Verona, 37134 Verona, Italy

**Keywords:** *Strongyloides stercoralis*, strongyloidiasis, migrants, diagnostic tests, ELISA, accuracy, prevalence

## Abstract

*Strongyloides stercoralis* infection is a life-threatening neglected tropical disease. Diagnostic issues have caused an underestimation of its global burden. The choice of appropriate diagnostic tests for the screening of populations at risk of the infection, such as migrants from endemic countries, is of paramount importance. From November 2017 to July 2018, all migrants presenting to the National Institute for Health Migration and Poverty (INMP) in Rome, Italy were offered screening tests for *S. stercoralis* infection. The study objective was to estimate the prevalence of strongyloidiasis in the study population and the accuracy of a novel ELISA assay. The following tests were carried out at the IRCCS Sacro Cuore Don Calabria hospital in Negrar, Verona: stool microscopy, real-time PCR for *S. stercoralis*, in-house immunofluorescence test (IFAT), a commercial ELISA assay (Bordier ELISA), and a novel ELISA assay (Euroimmun ELISA). A latent class analysis (LCA) model set up with test results, clinical variables, and eosinophilia indicated a prevalence around 7.5%, in line with previous findings. The sensitivity and the specificity of Euroimmun ELISA were 90.6% (95% CI 80.5–100) and 87.7% (95CI 84.5–91.0); these results indicate that the novel ELISA assay would be suitable for screening of migrants from endemic countries.

## 1. Introduction

*Strongyloides stercoralis* infection is a neglected tropical disease (NTD) affecting an estimated 640 million people worldwide [1]. The infection is transmitted through direct penetration of the skin by infective (filariform) larvae free-living in soil contaminated by human faeces. Thus, the transmission occurs in areas with inadequate sewage systems and hygienic conditions [2]. The larvae moult while migrating in the human body, and the adult female worm settles in the intestine, where it produces eggs. The newborn rhabditoid larvae hatch out of the eggs while still in the bowel and are shed with faeces. However, some of them moult into the filariform stage before leaving the body and thus can re-infect the host through rectal mucosa or perianal skin. This auto-infective cycle leads to a chronic infection, which can perpetuate through the years even in absence of re-exposure to an external infective source [2]. The infected individual can be either asymptomatic or complain of a series of unspecific symptoms, mainly involving skin and respiratory and gastrointestinal tracts [3]. Symptoms can be mild and intermittent but under some circumstances can worsen and manifest as severe conditions. This mainly happens in immunosuppressed individuals, for whom the disseminated infection, characterized by all parasite stages migrating all over the body, represents a therapeutic challenge and is life-threatening [2]. To prevent the risk of dissemination, screening and individual diagnosis of cases followed by proper treatment of the infection, which is based on a single dose of ivermectin in case of uncomplicated disease [4], are of paramount importance, even in asymptomatic individuals. Unfortunately, there is no gold standard for the diagnosis of strongyloidiasis, and this causes an underestimation of the burden of the infection and poses infected individuals at risk of misdiagnosis [3]. Indeed, microscopy examination of stool samples has very low sensitivity for the detection of *S. stercoralis*. Although its sensitivity was demonstrated to improve with the examination of multiple samples, it still remains disappointing [5]. Baermann method and agar plate culture have better performance but are seldom done because they are time-consuming and require parasitological skills that are not widely available in non-endemic settings [6]. Polymerase chain reaction (PCR) for *S. stercoralis* demonstrated good sensitivity and specificity, though it is not yet widely available outside referral laboratories [7]. At the moment, serological tests show the best sensitivity, although cross-reactions, in particular with filarial nematodes, can cause false positive results [8,9]. However, due to the potential harm posed by the infection and the good tolerability of ivermectin, overtreatment of possible false-positive cases should be preferred to missed diagnosis and treatment.

This study was part of the Tropical Neglected Diseases Project, “Strengthening the fight against Neglected Tropical Diseases in the migrant population through the use of medical devices” that was carried out from August 2016 to July 2018. The project was led by the Italian National Institute for Health Migration and Poverty (INMP) with the collaboration of the Department of Infectious Tropical Diseases and Microbiology (DITM) of the IRCCS Sacro Cuore Don Calabria hospital, as regards the diagnostic aspects. The general objective of the project was to strengthen the fight against NTDs in migrant populations from endemic areas living in Rome through an estimate of the prevalence and an evaluation of the epidemiological characteristics of some major NTDs in order to achieve early diagnosis and care of affected migrants.

In this work, we report the results of the screening for *S. stercoralis*. Primary objective was to estimate the prevalence of strongyloidiasis in this population, based on a combination of diagnostic tests. Secondary objective was to estimate the accuracy of a novel ELISA test (Euroimmun ELISA).

## 2. Materials and Methods

This was a cross-sectional study, which took place from November 2017 to July 2018.

Campaigns for promotion of the screening activities were conducted in order to disseminate information about selected NTDs (in addition to strongyloidiasis, targets of the campaign were schistosomiasis and Chagas disease) and about the free access to a dedicated INMP outpatient clinic.

### 2.1. Participants

Migrants of any age were offered the extended screening upon spontaneous presentation to the INMP outpatient clinic. Demographic information such as data concerning migration route, personal habits, and living conditions (economic and hygienic aspects) were collected. An infectious diseases consultant sought written informed consent, provided medical visit, and asked the participants to supply faecal and blood samples for *S. stercoralis* (and other NTDs based on epidemiological risk factors) testing. Information sheets and informed consent forms were available in Italian, Spanish, English, and French. Transcultural mediators were available for illiterate people and for those speaking other languages (Arabic, for instance). Parents’ or legal guardians’ consent was sought for minors. All consenting consecutive participants underwent the screening and were included in the analysis. Participants received a copy of the results of the test and were treated accordingly at the INMP clinic. Any positive test (including serology) constituted an eligibility criterion for treatment.

### 2.2. Test Methods

All biological samples were collected at the INMP. Aliquots were sent to the laboratory of San Camillo hospital, Rome for the full blood count. Other samples were sent to the DITM, where the following tests were carried out: microscopy examination of faeces concentrated by Ritchie’s modified method, a commercial ELISA for *S. stercoralis*, an in-house immunofluorescence test (IFAT), a novel ELISA kit, and real-time PCR for *S. stercoralis*. An ELISA test for *Schistosoma mansoni* (Bordier Affinity Products SA, Crissier, Switzerland) was also done at DITM. The laboratory staff performing the serological tests were blinded towards the results of the other tests.

The ELISA commercial kit (Bordier Affinity Products SA, Crissier, Switzerland) is based on somatic antigens from larvae of *Strongyloides ratti* [10]. A previous retrospective study estimated its sensitivity and specificity at 90.8% (95% CI 85.8–95.7) and 94.0% (95% CI 91.2–96.9), respectively [9]. The test was performed as per manufacturer’s instructions. A normalized optical density (OD) ratio was used to compare the results obtained in different sessions. A ratio ≥1 defined positive results.

IFAT was an in-house assay implemented at the DITM [11], where it is routinely used for screening and individual diagnosis. The assay is based on antigens from *S. stercoralis* filariform larvae obtained from faecal culture. Titres ≥ 1:20 are considered positive. The test previously demonstrated sensitivity 94.6% (95% CI 90.7–98.5) and specificity 87.4% (83.4–91.3) [9].

The ELISA performed at the DITM was a novel kit from Euroimmun. The assay is based on antigens of *Strongyloides papillosus* [12]. The test was performed per the manufacturer’s instructions. Results were classified in accordance with the package leaflet: ratio <0.8 = negative; ratio ≥ 0.8 < 1.1 = borderline; ratio ≥1 = positive.

The real-time PCR is based on Verweji’s method [13] and is used routinely at the DITM. Briefly, for DNA extraction, about 200 mg of faeces were suspended in 200 µL of phosphate-buffered saline containing 2% polyvinylpolypyrolidone (Sigma-Aldrich, Milan, Italy) and frozen overnight at −20 °C until the extraction. After thawing and boiling, the samples were run by an automated extractor instrument (Magnapure LC.2, Roche Diagnostics, Monza, Italy). The real-time assay was performed as described previously [13]. The amplification target was the small-subunit rRNA gene sequence for S. stercoralis. Appropriate positive and negative controls were included in all the experiments. As control for PCR inhibitors and amplification quality, the PhHV-1 control DNA was amplified with the appropriate primers/probe mix in the same reaction as S. stercoralis in multiplex PCR. The reactions, detection, and data analysis were performed with the CFX96 detection system (Bio-Rad Laboratories, Milan, Italy). In a previous retrospective study, the method demonstrated a sensitivity of 56.8% (95% CI 41.0–71.6) [14]. Specificity was considered virtually 100%.

### 2.3. Analysis

The sample size was based on a convenience sample constituted by all eligible participants who were enrolled during the study period. This was aimed to maximize the power and the generalizability of the study results.

Demographic and clinical data were summarized using descriptive statistics and measures of variability and precision. All parameters were reported with 95% confidence intervals (CI). For proportions, the exact Clopper–Pearson CI was computed.

Diagnostic test results were presented in contingency tables where patient’s disease status was inferred based on results of each single test, the composite reference standard, and also on probabilistic models using latent class analysis (LCA) [15].

Composite reference standard (CRS) is an alternative method used for assessing test accuracy using a combination of tests and was obtained by applying the following rule: if microscopy or PCR were positive or both IFAT and Bordier ELISA were positive, then CRS = positive; otherwise CRS = negative.

Data analysis was performed using SAS software, version 9.4 (SAS Institute, Inc., Cary, NC, USA). Statistical significance level was fixed at 0.05.

## 3. Results

In total, 650 participants received screening for *S. stercoralis*. Ages ranged from 8 to 73 years (median = 27, Q1 = 20, Q3 = 39), and most participants were male 463/640 (71.23%). Most participants originated from Africa (400 out of 650, 61.5%), while the remaining 38.5% came from Latin America. Overall, 318 out of 650 participants (48.92%) lived in rural areas in their country of origin; the proportion of individuals living in rural areas was higher between Africans (56.7%) than between Latin Americans (36.4%).

Symptoms were reported by 248 out of 650 (38.15%) subjects. Most frequent symptoms were pruritus and abdominal pain, reported by 206/425 (31.7%) and 180/650 (27.7%) participants, respectively. In the subgroup of participants from Africa, symptoms were reported by 226 out of 400 individuals (56.5%), while only 22 out of the 250 participants from Latin America (8.8%) had symptoms. Eosinophilia (defined as > 400 eosinophils/mcL) was detected in 102/425 (24%) individuals.

Only 635 participants were screened with real-time PCR, while the whole cohort was tested with each one of the other tests. The faecal-based tests produced the following results: 19 out of 635 (3%) participants had positive PCR, 21 out of 650 (3.2%) had positive stool microscopy. A total of 17 samples were positive to both tests. Overall, the two tests demonstrated excellent agreement (k coefficient: 0.84). A total of 18 out of the 400 participants from Africa (4.5%) had positive stool microscopy, compared to three out of 250 (1.2%) participants from Latin America. The proportion of PCR positive results was also higher for Africans (15 out of 399 individuals with the test result, 3.8%) than for Latin Americans ( four out of 236, 1.7%).

The overall numbers of samples positive to Bordier ELISA, Euroimmun ELISA, and IFAT were 60/646 (9.29%), 113/648 (17.44%), and 118/646 (18.27%), respectively. The agreement between Bordier ELISA and Euroimmun, Bordier ELISA and IFAT, and Euroimmun and IFAT was moderate in all cases, k coefficients being 0.5198, 0.4106, and 0.4887, respectively. The proportion of participants with positive serological tests was similar between the two subgroups: 20.3%, 15.8%, and 9.05% participants of African origin were positive to IFAT, Euroimmun ELISA, and Bordier ELISA, respectively. The proportions of participants from Latin America with a positive result to IFAT, Euroimmun ELISA, and Bordier ELISA were 14.9%, 20%, and 9.7%, respectively.

**Figure 1:**
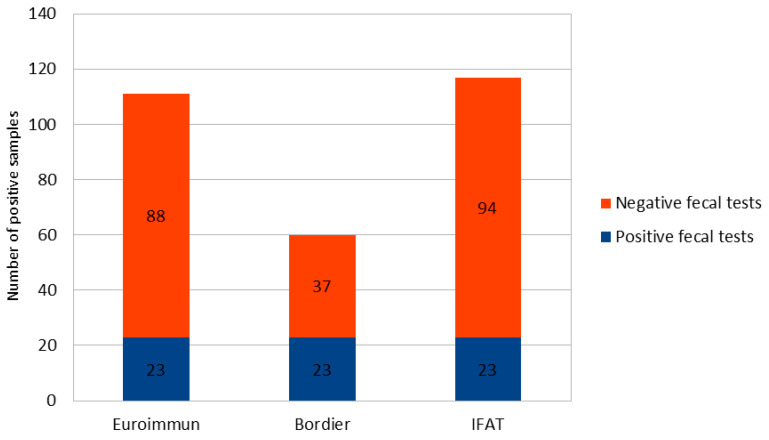
Positive samples to the serological tests compared to positive PCR/microscopy samples.

All 23 samples with positive PCR and/or microscopy were positive to all serological methods. Additional positive samples were found by the serological tests, as displayed in Figure 1.

According to the CRS, 43 samples were classified as positive and 605 as negative. Table 1 reports the number of positive and negative Euroimmun samples against this classification.

Sensitivity of Euroimmun ELISA was 93.2% (95%CI 80.9–98.5), specificity 87.9% (95% CI 85.1–90.4). Among the 73 false positive results (i.e., positive to Euroimmun ELISA but negative to the CRS), nine were found positive to hookworm and/or *Schistosoma* spp. Specifically, seven patients had positive ELISA, and three of them had also *S. mansoni* eggs in stools. Moreover, three patients had hookworm, one of them had also positive *S. mansoni* ELISA.

An LCA model using data from 425 participants for whom data on symptoms were available was set by adding to the results of all diagnostic tests (but Euroimmun ELISA), the clinical variables eosinophilia (*p*-value < 0.0001), and itching (*p*-value = 0.0072). Abdominal pain was excluded as it resulted statistically significant only in the univariable model (*p*-value = 0.0042). According to the LCA, 32 (7.5%) samples were classified as positive. Table 2 reports the number of positive and negative Euroimmun ELISA samples against this classification.

Based on these results, the sensitivity and the specificity of the Euroimmun ELISA were 90.6% (95%CI 80.5–100) and 87.7% (95%CI 84.5–91.0), respectively. The same helminthic co-infections (in terms of both type of helminths and number of cases) detected for the 73 false positives with the CRS were found among the 48 cases classified as false positives with the LCA.

## 4. Discussion

We collected data on screening for strongyloidiasis in a large cohort of immigrants from Africa (61.5%) and Latin America (38.5%). About one quarter of them presented eosinophilia, and 38% reported any symptom, mostly itching (31.7%) and abdominal pain (27.7%). About 3% of participants had a positive faecal test for *S. stercoralis* (PCR and/or stool microscopy), while the proportion of positive serological tests ranged from 9.3% of Bordier ELISA to 18.3% of IFAT.

The Euroimmun ELISA demonstrated good accuracy, with specificity around 88% according to both CRS and LCA, and a sensitivity of 93.2% (95%CI 80.9–98.5) with the CRS, decreasing to 90.6% (95%CI 80.5–100) with the LCA.

A systematic review [16] reported a 12.2% (95%CI 9.9–15.9) seroprevalence of strongyloidiasis in migrants living in the USA, Canada, Australia, New Zealand, Israel, and Western Europe. Our findings are in line with the review, although figures vary slightly based on the serological assay considered.

It should be considered that serology tends to overestimate the prevalence of strongyloidiasis based on the lower specificity compared to faecal-based tests caused by possible cross-reactions [9]. Here, we report a few co-helminthic infections that could be the cause of false positive results but, for instance, filarial nematodes were not thoroughly investigated. Although lower specificity might be acceptable in specific contexts (e.g., screen and treat strategies), a classification based on a combination of diagnostic tests can be more appropriate in others. As an example, surveys of prevalence in endemic areas can benefit from the addition of a more specific test, as co-infections with helminths that can cause cross-reactions are of higher concern in that setting. Here, we adopted two different approaches to the classification of positive/negative cases, which are CRS and LCA. With these methods, we classified as positive 6.6% and 7.5% cases, respectively. These figures, which are between the 3% found with faecal tests and a maximum of 18% with IFAT serology, can presumably give a better idea of the real prevalence of strongyloidiasis found in our cohort.

Eosinophilia and itching were included in the LCA on the basis of significant association with *S. stercoralis* infection in a multivariate analysis. Abdominal pain was significantly associated with the infection only in the univariate model, and other symptoms (for instance respiratory symptoms, weakness) did not show any association. Eosinophilia has repeatedly been reported as a frequent feature of strongyloidiasis, while clinical presentation is more debated, as symptoms are often intermittent and aspecific [17,18]. A systematic review of studies carried out in *Strongyloides* endemic areas [19] showed association with urticaria, while itching was frequent but the association was uncertain (though we might debate that itching is presumably associated with urticaria).

An unexpected result of our analysis is the excellent agreement between stool microscopy and PCR. Indeed, stool microscopy is usually considered to have a sensitivity exceedingly low compared to PCR [8]. The results of microscopy here (3.2% positive participants) are in line with a previous study reporting data on screening of 462 asylum seekers in Northern Italy [20], where 3.3% positive individuals were found with stool microscopy (with modified Ritchie’s concentration method). In other studies, the screening of populations at risk resulted in a higher proportion of individuals positive to *S. stercoralis* detected by PCR than other parasitological methods [6,21,22].

Here, it should be considered that laboratory staff performing microscopy was not blinded to the results of PCR, thus this might have caused a more intense examination of stool samples when PCR was positive. Conversely, agreement between the pair of serological assays was found good in all cases. In literature, we could find only a few conference proceedings reporting data on accuracy of Euroimmun ELISA, which was deemed good in comparison with other ELISAs [12,23]. Hence, to our knowledge, this is the first work reporting full data on the evaluation of this assay, which demonstrated good performance for the screening of individuals at risk of strongyloidiasis.

Finally, another limitation of the study is that prevalence of the infection was assessed in a group of migrants who spontaneously presented to the clinic. Thus, they might have been motivated by presence of symptoms or other health concerns, reducing the presence of asymptomatic infections in the cohort.

## 5. Conclusions

In a cohort of 650 individuals from Africa (61.5%) and Latin America (38.5%), screening with different diagnostic methods for *S. stercoralis* showed a prevalence of strongyloidiasis of about 7%. As expected, a higher proportion of positive results was found with serological tests than with faecal-based tests. Unexpectedly, Ritchie’s concentration stool microscopy showed excellent agreement with PCR for *S. stercoralis*. The accuracy of a novel ELISA (Euroimmun) kit was found good, indicating that the assay would be useful for screening activities.

## Figures and Tables

**Table 1:** Proportion of positive and negative results to the Euroimmun ELISA against the composite reference standard (CRS).

	Composite Reference Standard		
Euroimmun ELISA	Positive	Negative	Total
Positive	40	73	113
Negative	3	532	535
Total	43	605	648

**Table 2:** Results of Euroimmun ELISA according to the latent class analysis (LCA) classes.

	LCA Classes		
Euroimmun ELISA	Positive	Negative	Total
Positive	29	48	77
Negative	3	344	347
Total	32	392	648

## Data Availability

The study database will be available in Mendeley Data upon acceptance for publication.

## References

[1] Buonfrate D., Bisanzio D., Giorli G., Odermatt P., Fürst T., Greenaway C., French M., Reithinger R., Gobbi F., Montresor A. (2020). The Global Prevalence of Strongyloides stercoralis Infection. Pathogens.

[2] Nutman T.B. (2017). Human infection with Strongyloides stercoralis and other related Strongyloides species. Parasitology.

[3] Krolewiecki A.J., Lammie P., Jacobson J., Gabrielli A.F., Levecke B., Socias E., Arias L.M., Sosa N., Abraham D., Cimino R. (2013). A public health response against Strongyloides stercoralis: Time to look at soil-transmitted helminthiasis in full. PLoS Negl. Trop. Dis..

[4] Buonfrate D., Salas-Coronas J., Muñoz J., Maruri B.T., Rodari P., Castelli F., Zammarchi L., Bianchi L., Gobbi F., Cabezas-Fernández T. (2019). Multiple-dose versus single-dose ivermectin for Strongyloides stercoralis infection (Strong Treat 1 to 4): A multicentre, open-label, phase 3, randomised controlled superiority trial. Lancet Infect. Dis..

[5] Nielsen P.B., Mojon M. (1987). Improved diagnosis of strongyloides stercoralis by seven consecutive stool specimens. Zent. Fur Bakteriol. Mikrobiol. Und Hygiene. Ser. Amedical. Microbiol. Infect. Dis. Virol. Parasitol..

[6] Campo-Polanco L.F., Sarmiento J.M.H., Mesa M.A., Franco C.J.V., López L., Botero L.E., Builes L.A.G. (2018). Strongyloidiasis in humans: Diagnostic efficacy of four conventional methods and real-time polymerase chain reaction. Rev. Soc. Bras. Med. Trop..

[7] Buonfrate D., Requena-Mendez A., Angheben A., Cinquini M., Cruciani M., Fittipaldo A., Giorli G., Gobbi F., Piubelli C., Bisoffi Z. (2018). Accuracy of molecular biology techniques for the diagnosis of Strongyloides stercoralis infection-A systematic review and meta-analysis. PLoS Negl. Trop. Dis..

[8] Buonfrate D., Formenti F., Perandin F., Bisoffi Z. (2015). Novel approaches to the diagnosis of Strongyloides stercoralis infection. Clin. Microbiol. Infect. Off. Publ. Eur. Soc. Clin. Microbiol. Infect. Dis..

[9] Bisoffi Z., Buonfrate D., Sequi M., Mejia R., Cimino R.O., Krolewiecki A.J., Albonico M., Gobbo M., Bonafini S., Angheben A. (2014). Diagnostic accuracy of five serologic tests for Strongyloides stercoralis infection. PLoS Negl. Trop. Dis..

[10] van Doorn H.R., Koelewijn R., Hofwegen H., Gilis H., Wetsteyn J.C., Wismans P.J., Sarfati C., Vervoort T., van Gool T. (2007). Use of enzyme-linked immunosorbent assay and dipstick assay for detection of Strongyloides stercoralis infection in humans. J. Clin. Microbiol..

[11] Boscolo M., Gobbo M., Mantovani W., Degani M., Anselmi M., Monteiro G.B., Marocco S., Angheben A., Mistretta M., Santacatterina M. (2007). Evaluation of an indirect immunofluorescence assay for strongyloidiasis as a tool for diagnosis and follow-up. Clin. Vaccine Immunol. Cvi..

[12] Oesterreich B. Comparison of antigens from Strongyloides papillosus versus S. ratti for diagnosis of human strongyloidiasis by ELISA. Proceedings of the 2nd International Conference on Parasitology.

[13] Verweij J.J., Canales M., Polman K., Ziem J., Brienen E.A., Polderman A.M., van Lieshout L. (2009). Molecular diagnosis of Strongyloides stercoralis in faecal samples using real-time PCR. Trans. R. Soc. Trop. Med. Hyg..

[14] Buonfrate D., Perandin F., Formenti F., Bisoffi Z. (2017). A retrospective study comparing agar plate culture, indirect immunofluorescence and real-time PCR for the diagnosis of Strongyloides stercoralis infection. Parasitology.

[15] Andersen R., Hagenaars J.A., McCutcheon A.L. (2003). Applied Latent Class Analysis. Can. J. Sociol..

[16] Asundi A., Beliavsky A., Liu X.J., Akaberi A., Schwarzer G., Bisoffi Z., Requena-Méndez A., Shrier I., Greenaway C. (2019). Prevalence of strongyloidiasis and schistosomiasis among migrants: A systematic review and meta-analysis. Lancet Glob. Health.

[17] Martinez-Pérez A., Soriano-Pérez M.J., Salvador F., Gomez-Junyent J., Villar-Garcia J., Santin M., Muñoz C., González-Cordón A., Salas-Coronas J., Sulleiro E. (2020). Clinical Features Associated with Strongyloidiasis in Migrants and the Potential Impact of Immunosuppression: A Case Control Study. Pathogens.

[18] Ming D.K., Armstrong M., Lowe P., Chiodini P.L., Doherty J.F., Whitty C.J.M., McGregor A.C. (2019). Clinical and Diagnostic Features of 413 Patients Treated for Imported Strongyloidiasis at the Hospital for Tropical Diseases, London. Am. J. Trop. Med. Hyg..

[19] Tamarozzi F., Martello E., Giorli G., Fittipaldo A., Staffolani S., Montresor A., Bisoffi Z., Buonfrate D. (2019). Morbidity Associated with Chronic Strongyloides stercoralis Infection: A Systematic Review and Meta-Analysis. Am. J. Trop. Med. Hyg..

[20] Buonfrate D., Gobbi F., Marchese V., Postiglione C., Badona Monteiro G., Giorli G., Napoletano G., Bisoffi Z. (2018). Extended screening for infectious diseases among newly-arrived asylum seekers from Africa and Asia, Verona province, Italy, April 2014 to June 2015. Euro Surveill. Bull. Eur. Sur Les Mal. Transm. Eur. Commun. Dis. Bull..

[21] Amor A., Rodriguez E., Saugar J.M., Arroyo A., López-Quintana B., Abera B., Yimer M., Yizengaw E., Zewdie D., Ayehubizu Z. (2016). High prevalence of Strongyloides stercoralis in school-aged children in a rural highland of north-western Ethiopia: The role of intensive diagnostic work-up. Parasites Vectors.

[22] Becker S.L., Piraisoody N., Kramme S., Marti H., Silué K.D., Panning M., Nickel B., Kern W.V., Herrmann M., Hatz C.F. (2015). Real-time PCR for detection of Strongyloides stercoralis in human stool samples from Côte d’Ivoire: Diagnostic accuracy, inter-laboratory comparison and patterns of hookworm co-infection. Acta Trop..

[23] Warnecke J.M. Sensitive and specific ELISA for the serological diagnosis of Strongyloides infections. Proceedings of the 6th International Conference on Tropical Medicine and Infectious Diseases.

